# Outpatient chemotherapy with gemcitabine and oxaliplatin in patients with biliary tract cancer

**DOI:** 10.1038/sj.bjc.6603334

**Published:** 2006-09-12

**Authors:** J Harder, B Riecken, O Kummer, C Lohrmann, F Otto, H Usadel, M Geissler, O Opitz, H Henß

**Affiliations:** 1 Department of Gastroenterology and Hepatology, Freiburg University Hospital, Hugstetterstr. 55, Freiburg D-79106, Germany; 2 Department of Radiology, Freiburg University Hospital, Freiburg, Germany; 3 Department of Hematology and Oncology, Freiburg, Germany

**Keywords:** biliary tract neoplasms, chemotherapy, gemcitabine, oxaliplatin

## Abstract

This phase II study was conducted to determine the efficacy and toxicity of a gemcitabine (GEM) and oxaliplatin (OX) chemotherapy protocol in patients with unresectable biliary tract cancer (BTC). Patients were treated with GEM 1000 mg m^−2^ (30 min infusion) on days 1, 8, 15, and OX 100 mg m^−2^ (2 h infusion) on days 1 and 15 (gemcitabine and oxaliplatin (GEMOX-3 protocol), repeated every 28 days. The data were collected according to the Simon 2-stage design for a single centre phase II study (*α*=0.05; *β*=0.2). Primary end point was response rate; secondary end points were time-to-progression (TTP), median survival, and safety profile. Thirty-one patients were enrolled in the study between July 2002 and April 2005. Therapeutic responses were as follows: partial response in eight patients (26%, 95% confidence interval (CI) 14–44), stable disease in 14 patients (45%, 95%CI 29–62), resulting in a disease control rate of 71%. Nine patients (29%, 95%CI 16–47) had progressive disease. Median TTP was 6.5 months. Median overall survival was 11 months. Common Toxicity Criteria (CTC) Grade 3–4 toxicities were transient thrombocytopenia (23%), peripheral sensory neuropathy (19%), leucopenia (16%), and anaemia (10%). In conclusion the GEMOX-3 protocol is active and well tolerated in patients with advanced BTC. It can be applied in an outpatient setting with three visits per month only.

Biliary tract cancer (BTC) is a heterogeneous tumour entity with a dismal prognosis. Incidence and mortality rates of intrahepatic cholangiocarcinomas are rising, whereas gall bladder and extrahepatic cholangiocarcinoma incidences are slightly declining (Patel, 2001, 2002; Khan *et al*, 2002). At present, surgery is the only curative treatment option for BTC. However, less than 25% of patients are resectable at presentation with high relapse rates after surgery (Oertli *et al*, 1993; de Groen *et al*, 1999). The 5-year survival rates after resection for intrahepatic cholangiocarcinomas vary from 8–47% (Nakeeb *et al*, 2002).

A benefit over no therapy or best supportive care has been demonstrated for palliative treatment options with either surgical or endoscopic biliary drainage, and/or chemotherapy (Farley *et al*, 1995; Chang *et al*, 1998). However, no standard chemotherapy exists, owing to the rarity, and heterogeneity of the disease.

In recent phase-II trials, gemcitabine (GEM), a pyrimidine analogue, has been shown to be an active single agent therapy in BTC. For this drug objective response rates (RR) in a range from 8 to 60% have been reported (Raderer *et al*, 1999; Valencak *et al*, 1999; Gallardo *et al*, 2001; Gebbia *et al*, 2001; Kubicka *et al*, 2001; Penz *et al*, 2001). In addition, various combination therapies have been investigated in the setting of phase II studies with RRs between 9.5 and 53% (Gebbia *et al*, 2001; Patt *et al*, 2001; Doval *et al*, 2002; Kuhn *et al*, 2002; Nehls *et al*, 2002; Taieb *et al*, 2002; Kornek *et al*, 2004; Patt *et al*, 2004; Alberts *et al*, 2005; Ducreux *et al*, 2005). Overall, most favourable RRs in BTC were reached with protocols that combine cisplatin (CDDP) with 5-fluorouracil or GEM (Doval *et al*, 2002; Taieb *et al*, 2002; Ducreux *et al*, 2005).

Another combination regimen, which replaces CDDP by the third generation platinum analogue oxaliplatin (OX) could help to reduce the emetic and potential renal toxicity of CDDP without loss of treatment efficacy (Extra *et al*, 1998). A French study used the combination of GEM and OX in pancreatic cancer with promising results. Because of some similarities in tumour biology and response to chemotherapeutic agents between pancreatic and BTC we reasoned that gemcitabine and oxaliplatin (GEMOX) might be active in BTC as well. Therefore, the rationale for our study was to design a patient friendly protocol, with two in other cancers well tolerated and active substances. In the study by Louvet *et al* (2002) the two substances were given on 2 consecutive days with relatively long infusion times (fixed dose rate) for GEM. In contrast, we chose to apply GEM as an infusion over 30 min followed by OX as a 2 h infusion on the same day in an outpatient setting.

## PATIENTS AND METHODS

### Patients

Patients included in the study were required to meet the following criteria: 18–75 years old, written informed consent, histologically or cytologically confirmed and non-resectable or metastatic BTC, one or more bidimensionally measurable lesions on computed tomography (CT) or magnetic resonance imaging (MRI), Karnofsky Score ⩾70%, leucocyte ⩾3 × 10^9^ l^−1^, platelets ⩾100 × 10^9^ l^−1^, serum creatinine <2 mg dl^−1^, and bilirubin <3 mg dl^−1^, a life expectancy over 3 months, absence of cholangitis or carcinoma of the ampulla of Vater. Primary gastrointestinal cancers other than BTC were excluded by upper endoscopy, colonoscopy, and multislice CT scan. Women were either postmenopausal or were using adequate contraception with a negative pregnancy test at study entry. Patients were excluded in case of preexisting peripheral neuropathy National Cancer Institute-Common Toxicity Criteria (NCI CTC)>grade 1, use of major surgery or chemotherapy within 1 month, or radiotherapy within 12 months of study entry. Patients were allowed to have received prior chemotherapy (not including GEM or OX) for advanced disease or in the adjuvant setting. The baseline characteristics at study entry are given in Table 1. Patients with severe cardiovascular or pulmonary disease (NYHA III-IV, global respiratory insufficiency), symptomatic cerebral metastases, incompatibility with or allergy to platinol derivates, pregnancy, or lactation, serious infection, additional malignancies other than completely excised *in situ* carcinoma of the cervix or non-melanomatous skin cancer, and current alcohol or drug addiction were also excluded from the study. The study was carried out according to the Declaration of Helsinki Principles, the Good Clinical Practice criteria and was approved by the local ethics committee. The study was performed without sponsoring.

### Treatment plan

All patients received the treatment protocol GEMOX-3 on an outpatient basis as follows: first GEM 1000 mg m^−2^ as infusion over 30 min on days 1, 8, and 15, thereafter OX 100 mg m^−2^ over 2 h on days 1 and 15. Treatment was repeated every 28 days. NCI CTC version 2.0 was used for toxicity assessment. In addition a separate neurotoxicity scale for the assesment of OX-induced sensory neuropathy was used (Extra *et al*, 1998). Clinical examination including measurement of sensory neuropathy, blood count, and liver function tests were performed with each visit. In case of specific cumulative peripheral sensory neuropathy NCI CTC>grade 1 persisting over 7 days, the dose of OX was reduced to 75 mg m^−2^. Oxaliplatin was stopped in case of grade 3 or 4 peripheral sensory neuropathy. Treatment was subsequently continued according to the same schedule, but with GEM alone. With leucocytes <3.0 × 10^9^ l^−1^ or platelet count <100 × 10^9^ l^−1^ treatment was interrupted for 7 days. These criteria were applied to any day of treatment administration. If recovery occurred within the 7 days, treatment was continued without dose reduction, otherwise the dosages of GEM and OX were reduced to 75%. Doses omitted were given within the two subsequent weeks. After treatment discontinuation for more than 3 weeks the patients went off study. Treatment was continued until disease progression, unacceptable toxicity despite dose modification or patient withdrawal.

### Treatment evaluation and statistics

The data were collected according to Simon's optimal two-stage design for a single centre study (Simon, 1989). According to this design, an additional 19 patients were to be enrolled in case of a minimum RR of 10% in the first 10 patients. Thus, the planned minimum sample size was 29. In case of more than three responses in the total of 29 patients (RR of at least 10%), the regimen was considered active with *α*=0.05; *β*=0.2. All enrolled patients were included in the intention-to-treat analysis.

The primary end point was tumour response according the response evaluation criteria in solid tumours guidelines (Therasse *et al*, 2000). The response was specified in percent with 95%-CI calculated by the modified Wald method (Agresti, 1998). Secondary end points were time-to-progression (TTP), overall survival (OS), and toxicity. Time-to-progression was determined from the first day of treatment until tumour progression assessed by CT scan or MRI. OS was determined from first day of treatment until death. Time-to-progression and OS data were analysed by means of the Kaplan–Meier method.

## RESULTS

### Patient characteristics

The median duration of follow up was 13 months. The cutoff date for data analysis was 21 December 2005. Thirty-one consecutive patients at the Department of Medicine of the University of Freiburg, Germany, were enrolled from July 2002 to April 2005. Demographics and other baseline characteristics are summarised in Table 1. The study included 13 men and 18 women with a median age of 63 years (38–75). The primary cancer site has been: 14 (45%) intrahepatic bile ducts, 10 (32%) gallbladder, and 7 (23%) extrahepatic bile ducts. The baseline characteristics are similar to other phase II studies reported. Ampullary carcinomas were excluded.

### Safety and response

A total of 140 cycles of chemotherapy (420 treatment days) were delivered during the study. The median number of cycles was 4 (range 0.66–10). Three patients did not complete the first two cycles and were considered as progressive disease. One patient died of duodenal perforation owing to tumour infiltration without having completed the first cycle (received d1 and d8 only). In another two patients, chemotherapy was stopped after 1.66 cycles because of malignant biliary obstruction with consecutive development of an intrahepatic abscess and a cholangitis, respectively. Despite the absence of neutropenia, the immediate start of an appropriate antibiotic treatment, and adequate biliary drainage, both patients subsequently died of cholangiosepsis. Deaths in both patients had been attributed to tumour progression, rather than treatment related, although the latter could not completely be ruled out. All 31 patients were assessable for toxicity. Treatment delays of any reason were necessary in 27 out of 31 patients (87%). In 13 (42%) patients dose reductions had to be performed. In seven patients (23%), the dose of GEM and OX had to be reduced owing to bone marrow toxicity. In another six patients (19%), OX had to be reduced and later discontinued owing to peripheral sensory neuropathy. Sixty-six percent of treatment delays were caused by bone marrow toxicity, especially thrombocytopenia below 100 × 10^9^ l^−1^ (*n*=69/54%). Another major reason for delay of treatment were infections (9%), with febrile neutropenia in two patients. Toxicities according to NCI CTC are summarised in Table 2.

Despite these delays and dose reductions, eight patients (26, 95% CI 14–44%) had a partial response and 14 (45, 95% CI 29–62%) had stable disease. Thus, tumour control was achieved in 22 patients (71%). Median TTP was 6.4 month, whereas median OS was 11.0 month. Tumour or response re-evaluation was performed every other cycle by the same radiologic imaging (CT or MRI). Patients needed to have stable disease for a minimum of 8 weeks to be considered ‘disease control’.

Results are summarised in Table 3. Apart from the two patients mentioned above, there were no treatment-related deaths.

## DISCUSSION

Unresectable BTC is associated with a poor prognosis and treatment options are limited. As palliative therapeutic approach in patients with unresectable BTC we assessed the combination chemotherapy of GEMOX-3. Our data demonstrate that in advanced BTC the GEMOX-3 protocol shows good antitumour activity and tolerable toxicity with 3 treatment days per month and administration in an outpatient setting.

In palliative intention, novel combination chemotherapies are being tested in order to increase RRs and survival without additional toxicity. Although the combination of GEM and CDDP showed favourable RRs (RR 36.6%), the regimen was associated with a high frequency of grade 3 and 4 toxicities (Doval *et al*, 2004). At the start of the present study, two phase II trials have been published which analysed combinations with the new platinum component OX: GEM (fixed dose rate)/OX (RR 35.5%, PFS 5.7 months, median OS 15.4 months) (Andre *et al*, 2004), and capecitabine/OX (RR 26.6%, TTP NR, median OS NR) (Glover *et al*, 2005). Other combinations, for example, capecitabine/GEM, showed similar outcomes (RR 31%, PFS 7 months, OS 14 months) (Knox *et al*, 2005) The results of our study (RR 26%, TTP 6.4, OS 11.0) now underscore that GEMOX is an active combination chemotherapy with acceptable toxicity. The differences to the study of Andre *et al* (2004), might be explained by the inclusion of two groups of patients with different eligibility criteria in the French study (higher/lower bilirubin cutoff, better/worse performance status, previous chemotherapy). Although the group with stricter restrictions reached a RR of 35.5% and a superior OS of 15.4 months, the other group showed a RR of 22% and an inferior OS of 7.6 months. Longer infusion times of GEM (100 min *vs* 30 min) and the application of OX on a second day by Andre *et al* (2004) might also have influenced the outcome. In general, considering the short OS of patients with advanced BTC inpatient chemotherapy protocols should be minimised.

Thrombocytopenia was the most frequent toxicity in our study, but had no major clinical impact. The frequent treatment delays (54%) might be explained by the high thrombocyte cutoff level of 100 × 10^9^ l^−1^ chosen in the study and the cumulative toxicity of GEM and OX at the doses used. Another 9% of treatment delays were caused by infections of any kind, most frequently owing to cholangitis and occlusion of biliary stents in patients with extrahepatic and perihilar tumours. Despite this fact, patients with intraductal growth type BTC, tended to have a better OS than patients with mass forming type BTC (OS 13.3 *vs* 8.4).

With respect to neurotoxicity there was a clear relation between cumulative OX dose and delayed sensory neuropathy, as described in the literature (Extra *et al*, 1998; de Gramont *et al*, 2000). After therapy discontinuation, however, there was a fast improvement of treatment-related symptoms.

The important limitation of our study is its single centre phase II design. Owing to the limited number of patients, it was not possible to perform a randomised study comparing GEMOX to a single agent. However, a disease control>70% of patients suggests that patients with unresectable BTC benefit from the combination chemotherapy.

Some authors report an inferior RR in mass forming BTC (Nehls *et al*, 2003; Andre *et al*, 2004), whereas others document a more aggressive biology of gallbladder cancer (Doval *et al*, 2004, Knox *et al*, 2005). Despite the data are limited by the low number of patients it should be noted that patients with gallbladder cancer and extrahepatic BTC showed a considerably better RR (40 and 42%) than patients with intrahepatic BTC (RR 7%). This interesting observation should be verified in a larger trial that allows for a differentiated subgroup analysis.

Furthermore, despite extensive diagnostic efforts it cannot be ruled out that also patients with a cancer of unknown primary site were included and lead to a bias in the data. More precise molecular profile of the three BTC growth types will be necessary to better distinguish BTC from other solid tumours.

In summary, GEMOX-3 is efficacious and can be safely given on an outpatient basis as a palliative chemotherapy of advanced BTC. These promising RRs now need to be verified in controlled phase III trials, as they are currently ongoing.

## Figures and Tables

**Table 1:** Baseline characteristics of 31 patients with BTC

**Characteristic**	***N***	**(%)**
*Sex*		
Male	13	42
Female	18	58
		
*Age, years*		
Median	63	
Range	38–75	
		
*Karnofsky score*		
70	1	3
80	4	13
90	6	19
100	20	65
		
*Primary cancer site*		
Gallbladder	10	32
Intrahepatic bile ducts	14	45
Extrahepatic	7	23
		
*Disease at presentation*		
Locally advanced	3	10
Metastatic	28	90
		
*Metastatic sites*		
Liver	14	45
Lung	6	19
Lymph nodes	19	61
		
*Prior therapy*		
None	17	55
Surgery	7	23
Endoscopic biliary stenting	6	19
Radiochemotherapy and stent	1	3
Chemotherapy	0	0
		
*Presenting symptom*		
Pain	16	52
Jaundice	5	16
Weight loss	4	13
None	2	6
Fever	2	6
Fatigue	2	6
Total	20	65
Endoscopic biliary stenting during chemotherapy	3	10

BTC, biliary tract cancer.

**Table 2:** Grade 3–4 NCI CTC toxicities (*n*=31) worst toxicity (all cycles) per patient

**Grade 3–4 toxicity**	***N***	**(%)**
Leucocytopenia (grade 3)	5	16
Febrile neutropenia	2	7
Thrombocytopenia	7	23
Anaemia (grade 3)	3	10
Vomiting	0	0
Diarrhoea	0	0
Peripheral sensory neuropathy	6	19
Mucositis	0	0

NCI CTC, National Cancer Institute Common Toxicity Criteria.

**Table 3:** Treatment efficacy (*n*=31)

	**Overall**		**Intrahepatic**		**Gallbladder**		**Extrahepatic**	
**Treatment response**	***N***	**% (95%-CI)**	***N***	**%**	***N***	**%**	***N***	**%**
	31	100	14	100	10	100	7	100
Complete response	0	0	0	0	0	0	0	0
Partial response	8	26 (14–44)	1	7	4	40	3	42
Stable disease	14	45 (29–62)	9	64	3	30	2	29
Progressive disease	9	29 (16–47)	4	29	3	30	2	29
								
Median TTP (months)	6.4		6.2		6.0		7.3	
								
Median OS (months)	11.0		8.4		11.1		13.3	

CI, confidence interval; OS, overall survival; TTP, time-to-progression.
